# Progress in the Synthesis and Application of Tellurium Nanomaterials

**DOI:** 10.3390/nano13142057

**Published:** 2023-07-12

**Authors:** Hongliang Zhu, Li Fan, Kaili Wang, Hao Liu, Jiawei Zhang, Shancheng Yan

**Affiliations:** 1 School of Materials Science and Engineering, Nanjing University of Posts and Telecommunications, Nanjing 210023, China; 1221066837@njupt.edu.en (H.Z.); 1221066836@njupt.edu.en (L.F.); 1220066316@njupt.edu.cn (J.Z.); 2 School of Integrated Circuit Science and Engineering, Nanjing University of Posts and Telecommunications, Nanjing 210023, China; 1222228419@njupt.edu.cn; 3 School of Geography and Biological Information, Nanjing University of Posts and Telecommunications, Nanjing 210023, China; 1021173615@njupt.edu.cn

**Keywords:** tellurium nanostructures, electronics, optoelectronic

## Abstract

In recent decades, low-dimensional nanodevices have shown great potential to extend Moore’s Law. The n-type semiconductors already have several candidate materials for semiconductors with high carrier transport and device performance, but the development of their p-type counterparts remains a challenge. As a p-type narrow bandgap semiconductor, tellurium nanostructure has outstanding electrical properties, controllable bandgap, and good environmental stability. With the addition of methods for synthesizing various emerging tellurium nanostructures with controllable size, shape, and structure, tellurium nanomaterials show great application prospects in next-generation electronics and optoelectronic devices. For tellurium-based nanomaterials, scanning electron microscopy and transmission electron microscopy are the main characterization methods for their morphology. In this paper, the controllable synthesis methods of different tellurium nanostructures are reviewed, and the latest progress in the application of tellurium nanostructures is summarized. The applications of tellurium nanostructures in electronics and optoelectronics, including field-effect transistors, photodetectors, and sensors, are highlighted. Finally, the future challenges, opportunities, and development directions of tellurium nanomaterials are prospected.

## 1. Introduction

Nanomaterials have been widely studied by researchers because of their lack of size and have shown an important position in many disciplines and research fields [1,2,3,4,5]. Size and shape are two key parameters of nanomaterials. Size determines the size of the specific surface area and the number of atoms in a single dimension, while shape controls the structure of nanocrystals; size and shape together control the physical and chemical properties of nanomaterial [6,7]. Compared with traditional three-dimensional (3D) materials, low-dimensional materials are endowed with unique physical and chemical properties with small sizes and special structures. This makes low-dimensional nanomaterials have great development potential in the fields of electronics, nonlinear optics, photoelectric conversion, magnetic transport, and biomedicine [8,9,10,11,12,13,14,15,16,17,18,19,20,21,22,23,24,25,26,27]. Especially in the field of high-performance electronic devices, nanomaterials have the advantages of controllable size, high mobility, and low energy consumption, which makes low-dimensional devices one of the most promising candidates for extending Moore’s Law [28,29,30,31]. In addition, in the field of optoelectronics, traditional high-performance low-dimensional materials such as graphene have excellent thermodynamic and transport properties, but their bandgap width limits their application in photodetectors [32,33,34]. Another material, black phosphorus, has the advantages of an adjustable bandgap, but its shortcomings, include difficulty in synthesis and poor environmental stability [35,36,37]. Therefore, the development of a material that is easy to synthesize, has good environmental stability, and has an adjustable bandgap for photodetectors is now in urgent demand.

Tellurium (Te) belongs to the group VI Chalcogenus family. As a new low-dimensional nanomaterial, Te exhibits excellent optical and electrical properties, which has attracted widespread interest [38,39,40]. One-dimensional (1D) Te nanomaterials such as nanowires exhibit excellent electrical properties and ultra-high hole mobility (more than 600 cm^2^ V^−1^ s^−1^) as typical p-type semiconductors, making them very suitable for the construction of electronic and optoelectronic devices [41,42]. Two-dimensional (2D) Te nanomaterials exhibit excellent environmental stability, and because the layers are stacked by van der Waals forces, their thickness can reach a single layer. The bandgap of tellurium is thickness dependent, which allows the bandgap from 0.31 eV to 1.26 eV, making 2D Te a popular material for constructing photodetectors for broadband detection [43,44,45,46]. Due to the low melting point of Te, general synthesis methods such as the hydrothermal method and molecular beam epitaxy method are usually controlled below 200 °C, which makes it very suitable for the construction of devices in the low-temperature field, such as flexible electronic devices and 3D vertical integration. The controllable synthesis of Te nanomaterials has always been the object of extensive attention of researchers, and for this reason, many researchers have reported synthetic strategies for synthesizing Te nanomaterials with different structures. Such as hydrothermal l methods, microwave-assisted synthesis, true air phase deposition, molecular beam epitaxy, thermal evaporation, liquid phase stripping, and other methods [46,47,48,49,50,51,52,53,54,55,56,57,58,59,60,61,62]. The theoretical modeling of molecular dynamics ab initio has guiding significance for the synthesis of tellurium-based nanomaterials [63,64].

In this paper, we first summarize the different synthetic methods of Te nanostructures (including nanoparticles, nanowires, nanotubes, 3D nanostructures, and even chiral nanostructures). In addition, we highlight the use of Te nanomaterials in electronics and optoelectronics, including field-effect transistors, photodetectors, and sensors. Finally, the current challenges and prospects of Te nanomaterials in the field of optoelectronics are discussed.

## 2. Controlled Synthesis of Te Nanostructures

### 2.1. Synthesis of Zero-Dimensional Te Nanostructures

**Figure 1:**
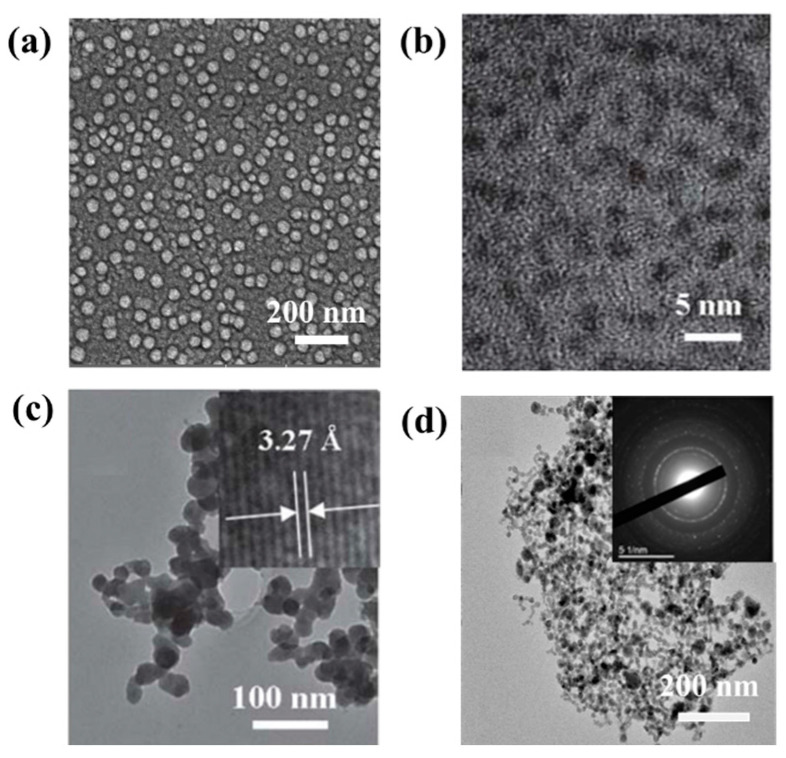
(**a**) SEM image of Te nanoparticles; (**b**) HTEM image of 1.5 nm Te nanoparticles; (**c**) HTEM image of 27.5 nm Te nanoparticles; (**d**) TEM images of the Te nanoparticles. Inset: diffraction patterns.

Zero-dimensional nanomaterials generally refer to nanostructures with a size of less than 1100 nm [65,66,67]. Yuan et al. [68] formed a simple redox reaction between NaHTe:NH_3_, NaH_2_Te, and ammonia solution by adding sodium borohydride (NaBH_4_) and ammonia (NH_4_OH) to the Te source with Te powder, resulting in a Te nanoparticle with an average diameter of 10 nm. Vahidi et al. [69] reported the extracellular synthesis of Te nanoparticles using the supernatant of Penicillium chrysogenum PTCC 5031, which has a spherical morphology with an average diameter of approximately 31 nm. The scanning electron microscopy (STM) of the resulting product is shown in Figure 1a. He et al. [70] reported a green synthesis method for Te nanoparticles at room temperature, which synthesized two different size distributions of Te nanoparticles using sodium telluride (Na_2_Te) as a Te source and oleic acid as an oxidizing agent with triethanolamine (TEA) dissolved in ethylene glycol (EG), one with a diameter concentrated at 1.5 − 0.5 nm and one with a diameter concentrated at 27.5 + 5 nm. Transmission electron microscopy (TEM) images of the two products are shown in Figure 1b,c. The oxidized Te is nucleated to produce Te nanoparticles with a diameter of 1.5 nm, which are then grown to 27.5 nm by the Ostwald maturation process. They are suspended in organic solvents and made into Te nanoparticle films with thicknesses of up to several hundred nanometers by electrophoretic deposition. Synthesizing zero-dimensional Te nanostructures in a simple and environmentally friendly manner remains a challenging problem. Guisbiers et al. [71] reported the first successful synthesis of pure Te nanoparticles by laser ablation in liquids. When different solvents are used, different sizes of products are obtained. Te nanoparticles larger than 100 nm in size are prepared in deionized water, and the size of the product becomes smaller when acetone is used as a solvent (Figure 1d).

### 2.2. Synthesis of 1D Te Nanostructures

In the past few decades, 1D materials such as nanotubes, nanowires, and nanoribbons have attracted people’s interest because of their unique structural, physical, and chemical properties [1,72,73]. Group VI Te has a unique chain-like structure; two separate Te atoms are linked by covalent bonds and grow spirally in the direction of the hexagonal basic unit parallel to [001] [55]. Te has a high-intensity anisotropy, and its 1D nanostructure has a tendency to grow in the direction of [001]. So far, various methods such as hydrothermal and solvothermal methods, microwave-assisted synthesis, thermal evaporation, vapor deposition, and dissolution recrystallization have been developed to achieve the synthesis of 1D Te nanomaterials, including nanowires, nanotubes, and nanoribbons [52,53,74,75].

#### 2.2.1. Synthesis of 1D Te Nanowires

**Figure 2:**
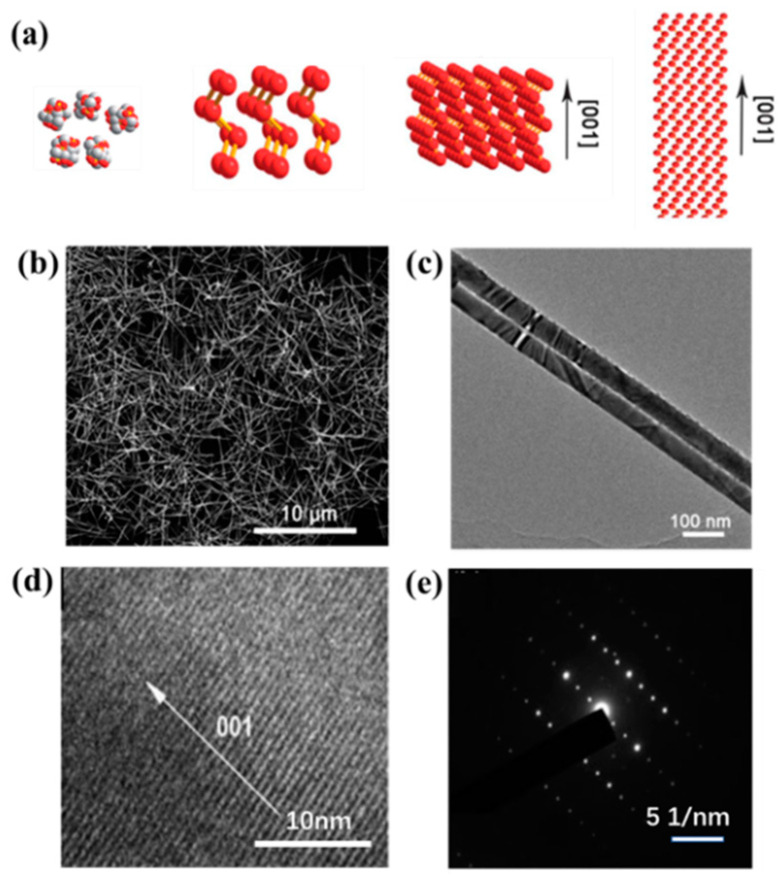
(**a**) Diagram of Te nanowire growth mechanism; (**b**) SEM image and (**c**) TEM image of Te nanowires; (**d**) By high-resolution transmission electron microscopy (HRTEM) image of Te nanowires, growing in the direction of extension [001]; (**e**) Te nanowires selective electron diffraction (SAED) image.

Te nanowires are a classical 1D nanostructure. The interesting and unique optical and electronic properties it exhibits have driven tremendous developments in the field of electronics and optoelectronic devices. Furuta et al. [76] synthesized Te whiskers by controlling the temperature of solid substrates using the vapor–solid method studied the growth path of Te whiskers, and found that substrate temperature and axial dislocation play a key role in whisker growth. Zhu et al. [55] developed a new microwave-assisted ionic liquid method for the rapid, controlled synthesis of Te nanorods and nanowires by combining the advantages of room-temperature ionic liquids and microwave heating. Li et al. [77] used EG acts as both a solvent and a reducing agent to synthesize Te nanowires in the presence of NaOH. The growth mechanism of Te nanowires was further studied, as shown in Figure 2a. Na_2_TeO_3_ is reduced to Te atoms and reacts for a long time under alkaline conditions to form crystal nuclei, and due to its highly anisotropic crystal structure, its preferential growth in the direction of extension [001] forms Te nanowires. Figure 2b,c shows the SEM image and the TEM image of Te nanowires. In order to achieve industrial application, Wang et al. [78] achieved the sub-kilogram-scale synthesis of Te nanowires by a pot of hydrothermal method. Up to 150 g of uniform Te nanowires with a diameter of 7–9 nm and a length of several microns can be synthesized. Hawley et al. [54] used physical vapor deposition to synthesize single-crystal Te nanowires with adjustable sizes using Te powder as a Te source in an inert atmosphere. These nanowires are about 1–22 μm in length and between 50 and 3000 nm in diameter. It was found that the morphology of Te nanowires was controlled by the reaction temperature, substrate temperature, and growth time. HRTEM and SAED patterns (Figure 2d,e) indicate that the synthesized Te nanowires have good crystallinity, and the growth of priority extension [001] is given. The two Te atoms are tightly bonded by covalent bonds, forming a helical chain structure. These helix chains are stacked by weak van der Waals interactions to form Te crystals. This would make it possible to separate Te crystals into single-atom chains. Recently, Qin et al. [79] eventually separated less-chain and single-chain Te nanowires by filling the cavity of carbon nanotubes (CNT) and boron nitride nanotubes (BNNT), respectively, by using physical vapor transport technology, respectively, by controlling the inner diameter of the CNT.

#### 2.2.2. Synthesis of 1D Te Nanotubes

**Figure 3:**
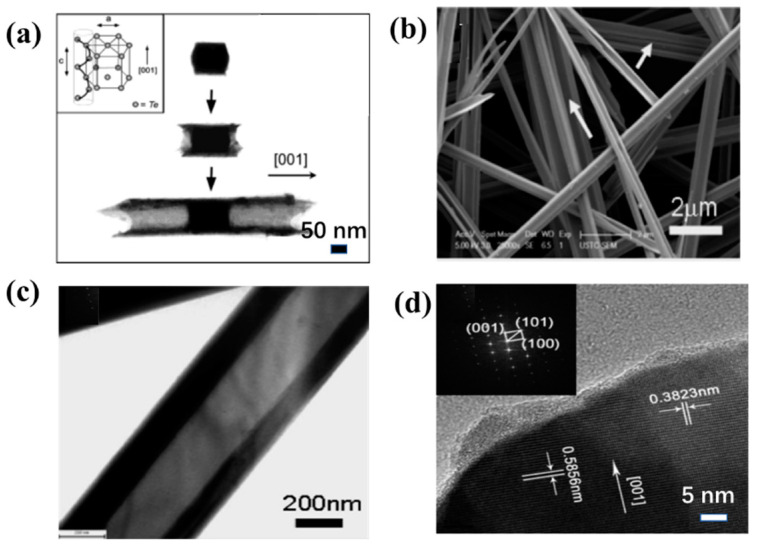
(**a**) TEM images of Te nanotubes at three different stages of growth; (**b**) TEM images of nanotube. As shown by the arrows in (**b**), some nanotubes are aggregated into bundles in solution or during the preparation of SEM samples; (**c**) FESEM image at high magnification; (**d**) HRTEM image of Te nanotubes.

One-dimensional hollow nanotubes are a classic 1D nanostructure because the existence of its inner surface structure has attracted widespread interest. Mayers et al. [80] synthesized Te nanotubes by liquid phase for the first using sodium tellurite as the Te source, ethylene glycol as the solvent and reducing agent, and then adding appropriate surfactants. The TEM image clearly shows the formation of concave seeds at both ends, followed by the growth of circumferential growth into nanotubes along the ends of the seeds (Figure 3a). They controlled the morphology of Te nanotubes by the molar amount of sodium tellurite. The concentration of sodium tellurite ranges from 40 mmol to 8 mmol to 0.7 mmol. As the concentration decreases, the diameter of cylindrical seeds ranges from about 260 to about 120 and to about 50 nm, respectively. At low concentrations of the Te source, when the diameter of the seed reaches a certain minimum value (possibly around 60 nm), the Te source in the central region of each seed is depleted and disappears, resulting in a morphological transition from hollow tubes to solid rods. Song et al. [81] selectively synthesized Te nanotubes with oblique cross-sections and hexagonal cross-sections by a surfactant-assisted solvothermal process. In the case of cetyltrimethylammonium bromide, Te nanotubes were synthesized with a length of 150–200 μm, an outer diameter of 100–500 nm, and a wall thickness of 50–100 nm (Figure 3b,c). When the surfactant is cellulose acetate, the synthetic Te nanotubes have a hexagonal cross-section. Mohanty et al. [82] synthesized single Te nanotubes with a triangular section by physical vapor deposition for the first time (Figure 3d). They found that the formation of nanotubes is highly dependent on the substrate material, gas flow rate, and deposition temperature. When Te powder is evaporated by heating at 350 °C and condensed downstream in an Ar atmosphere at a flow rate of 25 sccm on a Si (100) substrate for 10 min. This leads to the formation of nanotubes of triangular cross-sections, as well as some hexagonal nanotubes.

#### 2.2.3. Synthesis of 1D Te Nanoribbons

**Figure 4:**
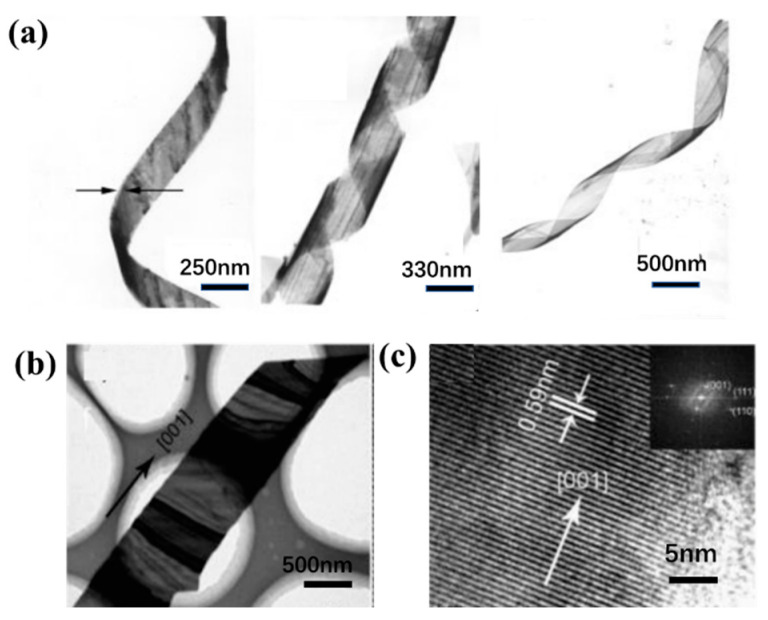
(**a**) A series of TEM images of Te nanoribbons in different distorted states; (**b**) TEM image and (**c**) HRTEM of the Te nanobelt.

Nanoribbons are 1D nanostructures with ribbon structures, and the synthesis of Te nanoribbons is usually achieved using hydrothermal methods and vapor deposition methods for liquid phase synthesis. Mo et al. [83] disproportioned sodium tellurite (Na_2_TeO_3_) in aqueous ammonia to generate nanoribbons with lengths of up to hundreds of microns. From the TEM image (Figure 4a), it can be seen that the generated nanoribbons have uniform thickness and good bending properties, and the growth kinetics of Te nanoribbons are controlled by temperature, pH, and reaction time play a decisive role in their formation. In addition to liquid phase synthesis, vapor deposition is also one of the important ways to generate Te nanoribbons. Wang et al. [59] prepared ultra-wide nanoribbons by placing Te powder into a vacuum system at an evaporation temperature of 350 °C and a deposition temperature of 449 °C (Figure 4b). They believed that in the Te crystal structure, there are spiral chains formed by covalently bonded atoms that are stacked together by weak van der Waals interactions to form hexagonal lattices. The anisotropic crystal structure of Te makes it grow from the z-axis. It is easy to form Te nanoribbons extending along the side of [001] (Figure 4c).

### 2.3. Synthesis of 2D Te Nanostructure

**Figure 5:**
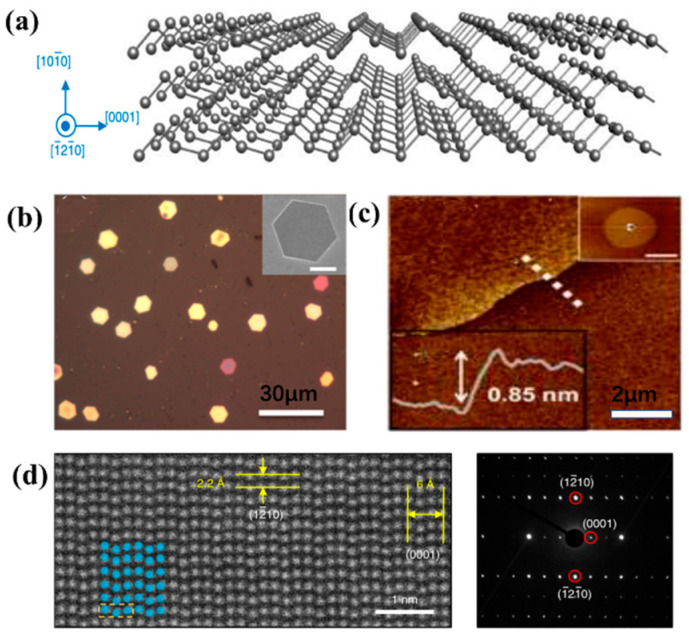
(**a**) Three-dimensional illustration of the structure of tellurene320; (**b**) Optical image of a 2D Te nanoplate. Inset: SEM image of a single nanoplate; (**c**) AFM images and cross-sectional images. Inset: magnification views and line profiles; (**d**) Telluene STEM image and SAED image.

Since the discovery of graphene, two-dimensional materials have attracted widespread attention with their unique structure and photoelectric properties [84,85]. Unlike zero-dimensional and 1D Te nanostructures, 2D tellurene has only slowly entered people’s field of vision in recent years. Te consists of chains of atoms in a triangular helix that is stacked together by van der Waals forces. When viewed along the x-axis, the zigzag layers are stacked together by van der Waals forces, as shown in Figure 5a, which is easier to break than the strong force between atoms, making it possible to synthesize fewer layers of 2D tellurene. The synthesis methods of 2D tellurene generally include physics-based van der Waals epitaxy (vdWE), Physical Vapor Deposition (PVD), Liquid-Phase Evolution (LPE), and chemistry-based liquid phase synthesis.

Van der Waals epitaxy has been recognized as a classical synthesis technique in the growth of 2D layered materials, which can overcome large lattice mismatches. Zhu et al. [86] first proceeded with vapor deposition on flexible and transparent fluor phlogopite sheets by vdWE [87]. Two-dimensional hexagonal Te nanoplates with good crystallinity crystals, large transverse size, and thin thickness were obtained (Figure 5b). Single-layer and few-layer two-dimensional structures show great potential in electronics and optoelectronic applications. Qiao et al. [88] used the particle swarm optimization method combined with first-principle density functional theory calculations to predict that a new class of 2D tellurene lesser layers can exist in stable 1T-MoS2-like (α-Te) structures and metastable tetragonal (β-Te) and 2H-MoS2-ike (γ-Te) structures, and calculated that the middle monolayer α-a and dβ-Te phases have higher carrier mobility. Subsequently, Zhu et al. [86] adopted the LPE method for the first time. The synthesized 2D Te nanosheets had a wide transverse size (41.5–177.5 nm) and a thickness from 5.1 to 6.4 nm. Apte et al. [89] used PVD to form ultra-thin Te sheets with a thickness of less than 7 nm and an area of 50 μm. They found that thermal evaporation of large blocks of Te in an Ar/H_2_ atmosphere at 650 °C led to the growth of ultrathin films on Si/SiO_2_ substrates and experimentally grew 1–3 layers of Te films (0.85 nm) relatively inexpensively (Figure 5c).

To achieve a large-scale, large-scale synthesis of 2D Te, Wang et al. [90] used a low-temperature, substrate-less solution process to fabricate large-area, high-quality 2D tellurene. They grew into 2D flakes with edge lengths of 50 to 100 μm and thicknesses of 10 to 100 nm by reducing Na_2_TeO_3_ in an alkaline solution by hydrazine hydrate (N_2_H_4_·H_2_O) at a temperature of 160 to 200 °C in the presence of the crystalline surface blocking ligand polyvinylpyrrolidone (PVP). Their team further studied the growth pathway of Te nanoribbons. They believed that the control of PVP concentration is the key to obtaining 2D Te. For each PVP concentration, the initial growth product is a predominantly 1D nanostructure, and the morphology of the growth product changes interestingly over time. After a certain period of reaction, structures with both 1D and 2D features begin to appear. Through TEM images and SAED (Figure 5d), it is found that these nanosheets grow in the direction of (0001) on the long axis (highlighting the 1D features of the nanostructure) and (1–210) in the lateral (highlighting the 2D features of the nanostructure), {1010} as the bottom and top surfaces of the nanoribbons. During initial growth, PVP is adsorbed on the {10–10} surface of the seed, which allows the product to form a 1D nanostructure. Growth along the {1–210} direction increases significantly due to assembly driven by thermodynamics. Enhanced growth along the {1–210} direction, as well as sustained (0001) growth, leads to the formation of 2D Te.

### 2.4. Synthesis of Three-Dimensional Te Nanostructures

**Figure 6:**
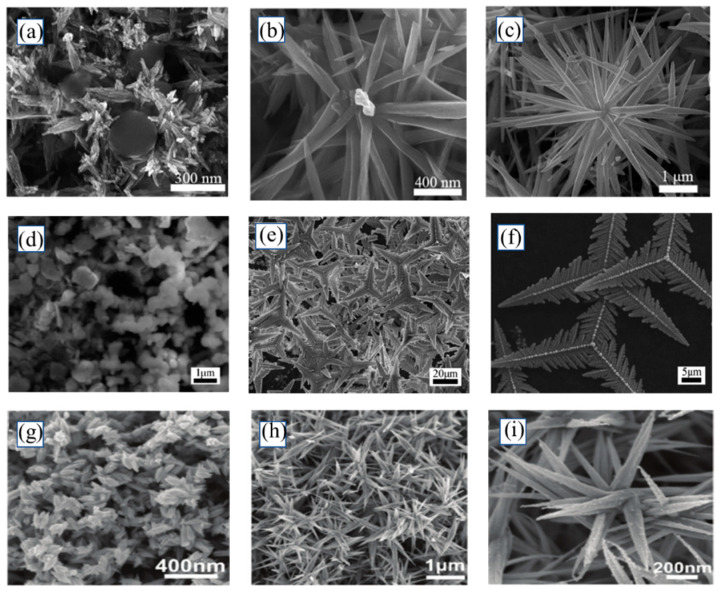
(**a**) SEM images of the produced at different reaction times: (**a**) 20, (**b**) 90, (**c**) 150 min. SEM images of Te produced at different reaction times: (**d**) 3, (**e**) 4, (**f**) 5 h. The reaction temperature was 130 °C. SEM images of obtained Te particles using N-methylformamide (**g**) or ethylene glycol (**h**,**i**).

The size and morphology of nanomaterials have a large impact on their properties and applications, and the synthesis of 3D complex structures is still a great challenge in the field of materials compared with 1D Te nanomaterials. To explore the synthesis and properties of 3D Te nanomaterials, Wang et al. [91] synthesized flower-like 3D Te nanostructures by a solvothermal method using diethyldithiocarbamate acid as the Te source and 2,2-dithiodibenzoic acid as the reducing agent. To explore the formation mechanism of products, products at different times were analyzed by SEM. The analysis demonstrates that the formation of Te nanoflowers is divided into three stages. The first stage is at the beginning of the initial reaction when the Te seeds produced are clustered together to form Te nanoclusters. In the second stage, these Te nanoclusters grow into nanorods. Due to their inherent high anisotropy, these nanorods attract Te atoms in solution, resulting in the continuous growth of flower-like superstructures in stage 3, which is through the Ostwald ripening process (Figure 6a–c). Their team synthesized gold-decorated tripod-shaped Te crystals by the hydrothermal method using the same Te source and reducing agent and found that the addition of ethylene glycol as a co-solvent was essential to obtain homogeneous triangular prism-shaped crystals (Figure 6d–f) [92]. Zhang et al. [93] dried trialkyl phosphonium telluride with common polar sub-solvents (e.g., water, alcohol, or amide) at high temperatures by the ionic liquid method. Te nanostructures with different morphologies were obtained, including 3D Te fusiform components and 3D aloe-like Te microstructures (Figure 6g–i). The authors further investigated the formation mechanism of 3D nanostructures. Commercially available [P6614] Cl typically contains about 0.1–0.5% HCl. It was found that the presence of HCl impurities in [P6614] Cl reduced the solubility of Te and played an important role in the formation of Te microstructure after the addition of polar proton solvents, and acidity could make Te bonds break rapidly, which promoted the precipitation of Te and the formation of Te crystals.

### 2.5. Chiral Te Nanostructures

**Figure 7:**
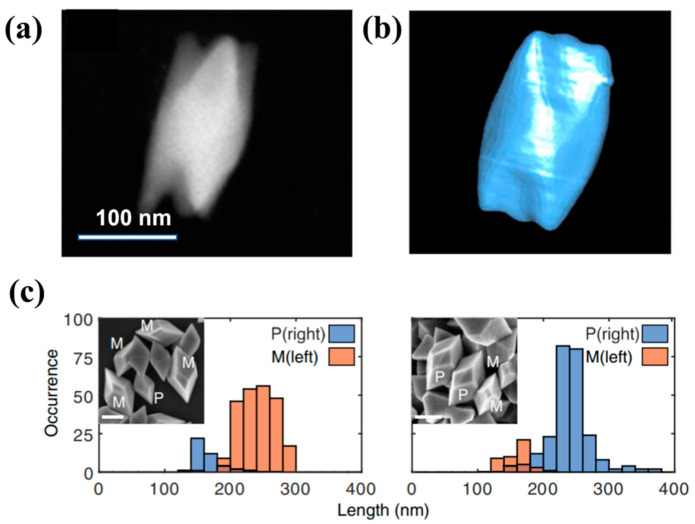
(**a**) STEM image and (**b**) Chromatographic reconstruction of chiral crystals; Distribution of bipyramid handedness when using only (**c**) L- or D-penicillamine ligands in the reaction (left and right, respectively). Orange and blue columns represent left- and right-handed particles, respectively. Bottoms of columns that appear brown are where orange and blue overlap. Insets: SEM images show the existence of both mirror images in each sample.

Chirality has always been a popular direction in the fields of materials science, chemistry, biomedicine, and physics because of its lack of symmetry [94,95,96,97,98,99]. Chiral nanomaterials exhibit many unique properties, such as enhanced circular dichroism and strong circular polarization luminescence, which have attracted widespread attention. How to controllably synthesize chiral nanocrystals has become a key challenge that may solve the physics of materials. Markovich et al. [100] reduced the Te precursor with hydrazine in the presence of large chiral biomolecules such as cysteine, penicillamine, and glutathione to synthesize a chiral-shaped Te nanostructure. Seen using a darkfield detector in STEM mode, their shape approximates triangular prisms, as shown in Figure 7a,b. The growth mechanism of these shapes was studied through TEM experiments, and the nanostructures appear to be formed by the coalescence of small clusters to form nanoparticles, which then form hexagonal tubular structures. This growth method conforms to the mechanism of “enantiomeric specific directional linkage” to grow chiral crystals, in which the initially formed chiral atomic clusters attach directionally to form chiral nanocrystals. Recently, Ben-Moshe et al. [101] reduced tellurium dioxide in the presence of chiral thiolated penicillamine ligands and reducing agent hydrazine, and synthesized chiral tellurium nanocrystals by blocking lateral growth by sodium lauryl sulfate (SDS). When added early in the reaction, the product is a twisted nanorod, and when SDS is added later, a thick triangular bipyramid is formed. When added early in the reaction, thin twisted nanorods are formed, while when SDS is added later, thick triangular bipyramids are formed. Bipyramidal nanoparticles were further characterized by SEM and STEM tomography, and 3D chiral morphology was resolved. The chiral crystals of Te were induced by left-handed and right-handed ligands, and it was found that chiral ligands had a great influence on the left and right chirality of Te crystals (Figure 7c).

## 3. Applications of Te Nanostructures

Te, as a typical p-type semiconductor, exhibits a bandgap of 0.35 eV at room temperature [102,103]. When the size is processed to the nanometer level, it exhibits many unique physical properties, such as high thermoelectric, piezoelectric characteristics, photoconductivity, and nonlinear optical response. This makes Te nanomaterials excellent candidates for applications such as field-effect transistors, photodetectors, and sensors [104,105,106].

### 3.1. Field-Effect Transistor

**Figure 8:**
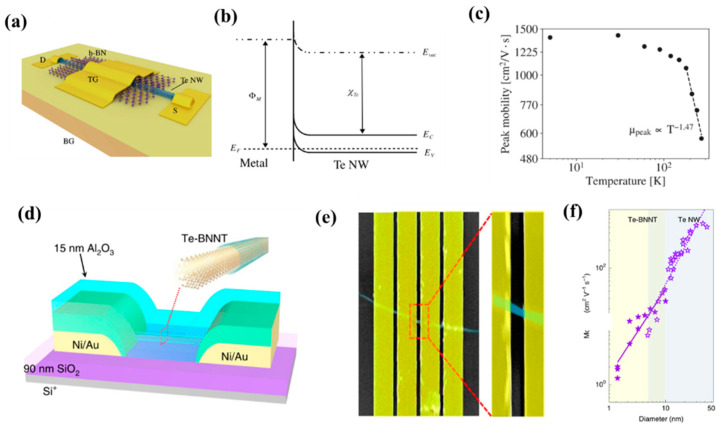
(**a**) Schematic diagram of a FET device; (**b**) Equilibrium band diagram of the device; (**c**) Hole mobility as a function of temperature dependence; (**d**) Schematic diagram of the Te-BNNT FET; (**e**) SEM image of the device; (**f**) carrier mobility at V_ds_ = 1 V of Te-BNNTs and Te nanowire short-channel FETs.

As an important device in integrated circuits, FETs have channel widths that limit their further integration development. To break through Moore’s Law, nanomaterials have gradually entered people’s field of vision, such as black phosphorus, graphene, molybdenum disulfide, and other materials have been widely used as the channel of the field-effect tube [107,108,109]. As a stable p-type semiconductor, Te can be synthesized on a large scale by green and low-cost methods. Qian et al. [110] synthesized Te nanowires using the hydrothermal method to study their electrical properties. They reported p-type semiconductor Te nanowires for the first time. For a typical Te nanowire FET, at room temperature, the calculated mobility of the Te FET at a bias voltage of −1 V is about 163 cm^2^ V^−1^ s^−1^, and the switching ratio exceeds 1000. As the transistor size shrinks, the device footprint decreases, and the effective contact area decreases. This causes the contact resistance and on-resistance of the channels and electrodes to increase, which negatively affects the overall drive current, switching speed, and power dissipation of the device. To address these issues, Majumdar et al. [41] proposed a double-gate Te nanowire FET (Figure 8a). They found that plasma cleaning is a critical step in the manufacturing process, helping to remove residual PVP overlays and possible interfacial oxides, further improving interface quality and reducing contact resistance. The qualitative band diagram of the metal (Ni)-Te junction in equilibrium is shown in Figure 8b, the Fermi level in Te is close to the valence band, and the Te nanowires are in contact with the metal electrode Ni as an ideal ohmic. The designed device has a hole mobility of 570 cm^2^ V^−1^ s^−1^ at 270 K, which increases to 1390 cm^2^ V^−1^ s^−1^ when the temperature drops further, and its switching ratio also exceeds 2 × 10^4^ (Figure 8c). To break through Moore’s Law on field-effect transistors, Qin et al. [79] separated a few strands and single-stranded Te nanowires from carbon nanotubes and boron nitride nanotube packages. Te nanowires encapsulated in boron nitride nanotubes were used to fabricate field-effect transistors with a diameter of only 2 nm. It was found that the current capability value of the Te-BNNT device reaches 1.5 × 10^8^ A cm^−2^, which was almost two orders of magnitude larger than that of the naked Te nanowire device (Figure 8d,e). The authors further analyzed the diameter dependence of the electrical properties of Te-BNNT devices and unpackaged Te nanowires. Packaged Te-BNNT devices continue to exhibit electrical performance with smaller diameter (approximately 2 nm) Te nanowires, whereas for unpackaged Te nanowires, the diameter must exceed 5 nm to exhibit electrical performance. Te nanowires exhibit excellent carrier mobility, greater than about 600 cm^2^ V^−1^ s^−1^ for diameters of 25 nm (Figure 8f). The decrease in mobility in narrower samples can be attributed to surface oxidation and defects. Carrier mobility decreases with smaller diameter Te nanowires, with the average carrier mobility of 2 nm Te nanowires being about 1.85 cm^2^ V^−1^ s^−1^. In 2018, Wang et al. [90] synthesized 2D Te on a large scale by liquid phase for the first time and studied the transport characteristics of a 2D FET. Due to its narrow bandgap characteristics, the device exhibits p-type characteristics with slight bipolar transmission behavior. The authors further performed the thickness dependence of two key indicators of on/off ratio and field effect mobility through more than 50 devices with long channel devices of 2D Te, and the thickness of Te ranges from 35 nm−0.5 nm (monolayer). The FET exhibits a switching ratio of 1 × 10^6^ when there are fewer layers and drops sharply to 10 as the thickness increases, which may be caused by the decrease in gate electrostatic control in the thicker sheets. At room temperature, the hole mobility of the device reaches a maximum of 700 cm^2^ V^−1^ s^−1^ at a Te nanosheet thickness of 16 nm and decreases to about 1 cm^2^ V^−1^ s^−1^ when the thickness is reduced to 1 nm (bilayer).

**Figure 9:**
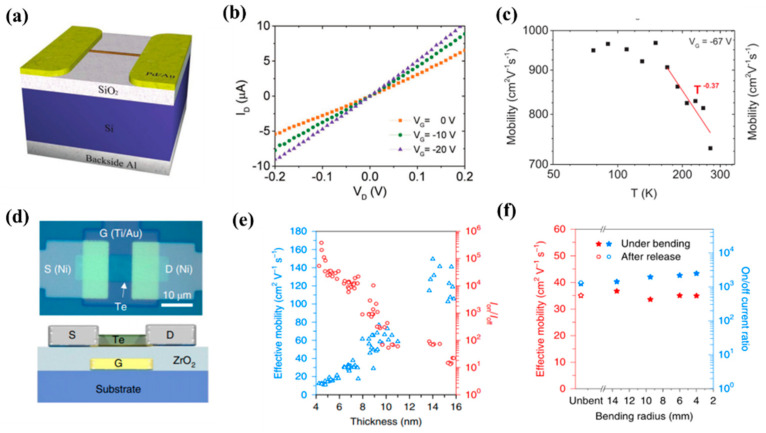
(**a**) Schematic of the device structure; (**b**) I_D_-V_D_ curve of the device; (**c**) The fit of the mobility of the device over the 180–300 K temperature range, shown by the red line; (**d**) Optical image and device structure schematic of Te FETs; (**e**) Thickness-dependent mobility (blue) and on/off current ratio (red) of Te FETs; (**f**) Effective mobility and on/off current ratio of Te FETs on Kapton substrates under different bending states.

Exploring semiconductors with high mobility grown at low temperatures (400 °C or less) is important for many applications, especially the flexible electronic devices and 3D vertical integration that is now attracting attention. Three-dimensional vertical integration, in which the devices and circuits directly grow and are manufactured on top of the silicon-based circuits and metal layers that have been manufactured, and their temperature should be strictly controlled below 450 °C to prevent damage to the underlying circuit. For another emerging flexible electronics technology, there are more stringent temperature limits (generally below 200 °C) because the manufacturing temperature needs to be maintained at the melting point or glass transition temperature of the substrate. However, cryogenic devices used in flexible electronics typically have very low carrier mobility. This limits the application of flexible devices. Zhou et al. [111] developed a device based on the helical Te nanostructure, and Figure 9a shows the schematic structure of the Te-based FET. It has room temperature field effect mobility of up to 707 cm^2^ V^−1^ s^−1^ and is grown directly by molecular beam epitaxy at low temperatures (≤120 °C) on substrates including SiO_2_/Si and polyethylene terephthalate (PET), which can be used for 3D monolithic integration or flexible electronics, respectively. Figure 9b shows a typical I_D_–V_D_ curve showing good ohmic contact. To investigate the potential carrier scattering mechanism, the temperature dependence of device mobility was investigated, as shown in Figure 9c, where mobility increases with decreasing temperature and reaches the maximum mobility of 965 cm^2^ V^−1^ s^−1^ at 90 K, indicating that mobility is phonon-limited. To realize the industrial application of Te FETs, Zhao et al. [112] obtained Te thin films at low temperatures by thermal evaporation to manufacture high-performance wafer-level FETs (Figure 9d). The temperature will have a great influence on the performance of Te film. An FET was made from evaporated Te film (8 nm) in the range of −80 °C to 25 °C, and it was found that the Te film evaporated at −80 °C showed the best transport characteristics and the transistor exhibited an effective hole mobility of about 35 cm^2^ V^−1^ s^−1^, an on/off current ratio of about 10^4^, and a subthreshold swing of 108 mV dec^−1^ at room temperature (Figure 9e). Since Te can be easily deposited on a variety of substrates using low-temperature evaporation technology, the authors fabricated 8-nanometer-thick Te FETs on 4-inch quartz wafers and PET substrates. For the mechanical flexibility test of Te FETs on Kapton substrate, it was found that there was no significant change in device mobility and on/off current ratio when bent to a radius of 4 mm or corresponding to tensile strain below 0.63% (Figure 9f), indicating that Te thin film FET is widely used in flexible and transparent electronic devices.

### 3.2. Photodetector

**Figure 10:**
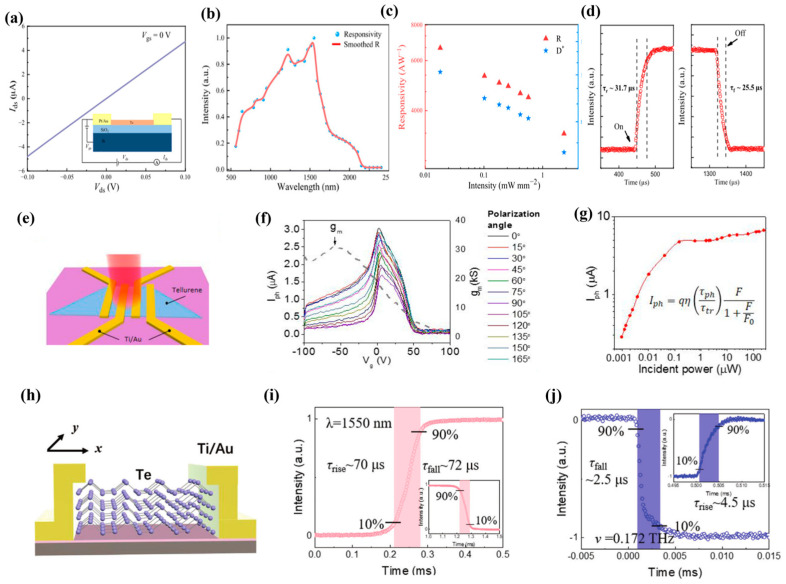
(**a**) V_ds_-I_ds_ curve of Te nanowire device. Inset: schematic diagram of the device; (**b**) At a bias of 0.1 V and a laser power intensity of 0.01 mW mm^−2^, light response spectra of Te nanowire devices (wavelengths from 500 to 2500 nm); (**c**) Under 1550 nm laser irradiation, the response rate and detection rate of Te nanowire devices are dependent on power intensity; (**d**) Time-resolved photoresponse of Te nanowires; (**e**) Schematic diagram of a Te device; (**f**) Photocurrent (left axis) and channel trans-conductance (right axis) measured at different polarization angles (V_ds_ = 1 V); (**g**) The power dependence of the photocurrent of the device; (**h**) Schematic diagram of Te detector structure; (**i**,**j**) Time-resolved photoresponse on IR (1550 nm) and THz (0.172 THz).

As a low-dimensional narrowband semiconductor, Te nanowires are widely used in infrared detectors due to their high hole mobility and small dark current. Zhong et al. [58] synthesized Te nanowires by hydrothermal method, measuring the electrical properties of the nanowires. At a temperature of 77 K, the photocurrent and responsivity of Te nanowire photodetectors are 0.17 mA and 25.8 A W^−1^ by excitation of infrared light at 980 nm, respectively. Then, the Te nanowires are assembled into a membrane, and the assembled detector exhibited a photocurrent of 26.6 μA and a responsivity of 86.52 AW^−1^. Peng et al. [113] synthesized highly crystalline Te nanowires by chemical vapor deposition and further studied their photoelectric properties. At room temperature, Te nanowire FET devices exhibit mobility in excess of 100 cm^2^ V^−1^ s^−1^ (Figure 10a). Te nanowire photodetectors are responsive to light at broadband wavelengths of 500 to 2500 nm and exhibit good responsivity in the wavelength range of 1100 to 1500 nm (Figure 10b). Under 1550 nm laser irradiation, the responsivity of the Te nanowire photodetectors is as high as 6650 AW^−1^. Compared to other state-of-the-art low-dimensional materials, its responsivity at 1550 nm is one of the best materials (BP and InGaSb responsivities of 3300 and 6000 AW^−1^, respectively, at a wavelength of 1550 nm). At a bias voltage of 0.1V, the specific detection rate of the Te nanowire photodetectors is as high as 1.23 × 10^12^ Jones (Figure 10c). At the same time, the rise and fall times of the Te nanowire device reached a staggering 31.7 and 25.5 μs, respectively (Figure 10d), representing one of the fastest speeds among previously reported infrared detectors. The responsiveness, response time, and detection capabilities of Te nanowire devices are impressive. The bandgap of two-dimensional Te can be widely used in broadband photodetectors through thickness adjustment and air stability. In 2018, Wang et al. [43] realized the large-scale synthesis of two-dimensional tellurene with adjustable thickness by liquid phase method, which aroused widespread interest among researchers. Qiao et al. [88] investigated the electrical properties of 2D Te by first principles. They found that 2D Te has high mobility and strong absorption in the visible and near-infrared wavelengths and predicted that it would be promising for photodetector applications. Immediately afterward, Shen et al. [43] synthesized Te nanosheets by hydrothermal method and used them for broadband and ultrasensitive photoelectric detection (Figure 10e). At room temperature, Te nanosheets showed hole mobility of up to 458 cm^2^V^−1^s^−1^, while Te photodetectors showed peak intrinsic response rates of 383 AW^−1^, 19.2 mAW^−1^, and 18.9 mAW^−1^ at wavelengths of 520 nm, 1.55 μm, and 3.39 μm, respectively. At wavelengths of 520 nm and 3.39 μm, the gains reach 1.9 × 10^3^ and 3.15 × 10^4^, respectively, due to the optical gating effect (Figure 10f,g). It is proved that the Te photodetectors achieve full coverage detection in the short infrared band. High-performance broadband photodetectors have important applications, including imaging, communications, and medicine, but achieving photodetectors covering visible (VIS), infrared (IR), terahertz (THz), and millimeter wave (MMW) bands remains a major challenge (Figure 10h). Huang et al. [114] grew high-quality Te nanosheets by PVD and proposed an ultra-broadband photodetector based on a metal–tellurium–metal structure that simultaneously covered visible, infrared, terahertz, and millimeter waves. In the VIS and IR bands, the incident light energy was greater than the bandgap, which changed the conductivity of the semiconductor due to the photoconductivity effect. The detector exhibited responsivities of 0.793 AW^−1^ and 9.38 AW^−1^ at 635 and 1550 nm. For the THz and MMW bands, the energy of the incident light was much lower than the bandgap of Te by 0.35 eV, and detection could only be achieved by another mechanism, the electromagnetic induction trap effect. The photodetectors exhibited high responsivities of 87.8 AW^−1^ and 986 AW^−1^ at 0.172 THz and 0.022 THz, respectively. Due to the different response mechanisms, the fast response time (≈4.5 μs) in the THZ band was an order of magnitude higher than that of detectors in the infrared band (e.g., 1550 nm) (Figure 10i,j).

### 3.3. Te Sensor

**Figure 11:**
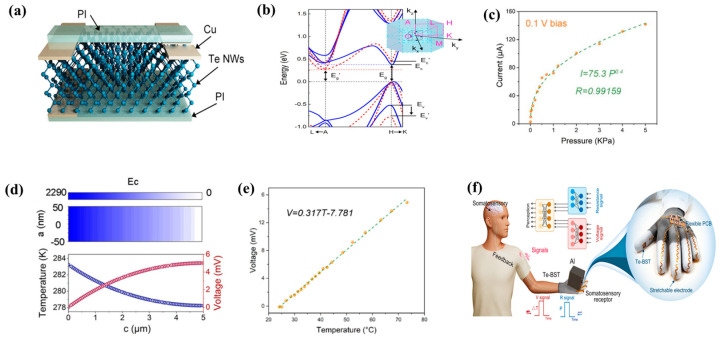
(**a**) Schematic diagram of bimodal tactile sensor; (**b**) Band structure of Te (**c**) At a bias voltage of 0.1 V, different pressures are applied and the current curve of the device is curved; (**d**) When there is a temperature difference between the two ends of Te nanowires, the potential of the device changes; (**e**) A curve of voltage as the temperature at the bottom of the device changes; (**f**) Schematic diagram of sensor operation.

Te is a chain-like structure at the atomic scale and has a high carrier transport rate and thermoelectric properties. Therefore, it has good electrical properties along the z-axis, which makes Te nanowires suitable for development into a state-of-the-art multifunctional sensor device. Li et al. [115] reported a bimodal sensor based on Te nanowires (Figure 11a). When pressure is applied to Te, the deformation causes its band structure to change. As shown in Figure 11b, when pressure is applied, the band structure of Te varies significantly along the z-axis. After the band widens, the band at point H becomes steeper, the effective carrier mass decreases, and the carrier mobility increases, thereby reducing the resistance. To test the performance of the device, its piezoresistive performance was evaluated, and the current increased from an initial 2.82 μA to 141.82 μA when the external pressure increased from 0 Pa to 5 kPa (Figure 11c). When the device is exposed to temperature stimuli, the temperature difference between the surface and the lower surface of the device causes a one-sided accumulation of carriers. For p-type semiconductors, most carrier holes accumulate on the low-temperature side, which gives it a higher potential. When the temperature is increased on one side of a Te nanowire device, the device generates a thermoelectric potential due to the Peltier effect (Figure 11d). To test the thermoelectric performance of the manufactured device, the thermal voltage associated with the temperature gradient of the device was measured. Previous studies have indicated that small temperature changes only affect the output voltage of the device and have a negligible effect on conductivity. As shown in Figure 11e, the output voltage increases linearly as the temperature increases over a small range. The above studies have proven that the device has good performance in temperature sensing and pressure sensing applications. In addition, the two signals do not interfere with each other, providing a reliable basis for the realization of bimodal sensing devices. Within the range of elastic strain of materials, pressure, and elastic strain become proportional to each other. Elastic strain reflects the human skin’s perception of the rigidity of the surface of the object, while thermal conductivity reflects the temperature of the surface of the object. Based on the above theory, a dual-mode tactile sensing device based on Te nanowires can sense the softness of the material and the surface temperature at the same time and realize the object recognition function (Figure 11f).

In addition to some of the above applications, the field of magnetoelectricity is also a hot research direction. Te forms a triangular crystal structure whose atoms are linked by covalent bonds to form a helical chain along the z-axis. Furukawa et al. [116] found that triangular Te crystals lack inverse symmetry, which leads to spin splitting in the bulk band and current-induced magnetization. It is worth noting that the direction of the current-induced magnetic field on the block Te is not circular but parallel to the applied current. This development opens a new field of electromagnetic induction.

Thermal stability has a certain influence on device performance. Zhao et al. [117] discussed the effect of annealing temperature on Te transistors. As the temperature increases, the performance of the Te device continues to deteriorate, and it fails at 200 °C. It is believed that the degradation mechanism of transistors is related to the degradation of channel material sublimation and contact. Through graphene contact and SiOx packaging, they increased the fault temperature of the device to 250 °C.

## 4. Summary and Outlook

In this paper, the synthesis methods of Te nanomaterials of different shapes and structures are discussed, and their applications in photoelectricity, such as field-effect transistors and photodetectors, are introduced. In the main liquid-phase synthesis, subtle changes in conditions play an important role in the control of Te nanostructures. The variation of the Te structure will have a great impact on its physical properties, such as the band gap width of 2D Te, depending on its thickness. Reasonable control of nanostructures will produce tremendous developments in the construction of nanodevices. At the same time, Te nanomaterials demonstrate versatile and potential applications for electronics, optoelectronics, piezoelectric and thermoelectric devices. Research on Te is likely to be in the following areas in the future: (1) Te nanostructure synthesis techniques should be optimized to synthesize large-size 2D Te and thin-walled Te nanotubes; (2) Te nanowires are grown directly on substrates and used for low-temperature 3D perpendicular integrated circuits; (3) Low-temperature synthesis, good ductility, and Te nanomaterials are used to design flexible wearable devices; (4) Te has emerged as a new type of magnetoelectric material. However, the application of Te nanomaterials in magnetoelectricity is still a challenge; (5) Chiral Te crystals have been successfully synthesized, and it is of great interest to try to synthesize new chiral crystals using Te crystals as a template. It is foreseeable that Te nanomaterials will have great prospects in the field of materials and optoelectronics.

## Data Availability

Not applicable.
